# Exploring recent trends (2014–21) in preferencing and accepting Queensland medical internships in rural hospitals

**DOI:** 10.1186/s12913-024-10683-z

**Published:** 2024-02-23

**Authors:** Matthew McGrail, Torres Woolley, Janani Pinidiyapathirage, Kath Paton, Deborah Smith, Kay Brumpton, Peta-Ann Teague

**Affiliations:** 1 The University of Queensland, Rural Clinical School, Rockhampton, QLD 4700 Australia; 2 James Cook University, College of Medicine & Dentistry, Townsville, QLD 4811 Australia; 3 Griffith University, School of Medicine and Dentistry, Southport, QLD 4222 Australia; 4 Rural Medical Education Australia, Toowoomba, QLD 4350 Australia

**Keywords:** Internship, Rural workforce, Medical training, Junior doctors, Rural immersion, Graduate medical education, Training pathways, Distribution

## Abstract

**Background:**

Medical internship is a key transition point in medical training from student to independent (junior) doctor. The national Regional Training Hubs (RTH) policy began across Australia in late 2017, which aims to build medical training pathways for junior doctors within a rural region and guide students, interns and trainees towards these. This study aims to explore preferencing and acceptance trends for rural medical internship positions in Queensland. Moreover, it focuses on internship preference and acceptance outcomes prior to and following the establishment of RTHs, and their association with key covariates such as rural training immersions offered by medical schools.

**Methods:**

Data from all applicants to Queensland Health intern positions between 2014–2021 were available, notably their preference order and location of accepted internship position, classified as rural or metropolitan. Matched data from Queensland’s medical schools were added for rural training time and other key demographics. Analyses explored the statistical associations between these factors and preferencing or accepting rural internships, comparing pre-RTH and post-RTH cohorts.

**Results:**

Domestic Queensland-trained graduates first preferencing rural intern positions increased significantly (pre-RTH 21.1% vs post-RTH 24.0%, *p* = 0.017), reinforced by a non-significant increase in rural acceptances (27.3% vs 29.7%, *p* = 0.070). Rural interns were more likely to have previously spent ≥ 11-weeks training in rural locations within medical school, be rurally based in the year applying for internship, or enrolled in the rural generalist pathway.

**Conclusions:**

The introduction of the RTH was associated with a moderate increase of graduates both preferencing and accepting a rural internship, though a richer understanding of the dominant reasons for and against this remain less clear. An expansion of graduates who undertook longer periods of undergraduate rural training in the same period did not diminish the proportion choosing a rural internship, suggesting there remains an appetite for these opportunities. Overall, domestic graduates are identified as a reliable source of intern recruitment and retention to rural hospitals across Queensland, with entry to the rural generalist pathway and extended rural placement experiences enhancing uptake of rural practice.

## Introduction

Geographic maldistribution of metropolitan and rural doctors persists world-wide. There are many underlying factors, including few medical schools producing rural doctors as central to their organisational mission, inadequate selection of students with rural backgrounds and/or rural interest, the growth and incompatibility with rural practice of some medical sub-specialisations, and many doctors remaining near to where they complete their training in larger cities, particularly beyond medical school [1, 2].

In most countries an internship immediately after graduation from medical school is required for gaining general registration, either before entry to specialty college training (as in Australia and the UK) or embedded within residency programs (as in the USA). Medical internship is the key transition point in the training pathway from medical student to independent (junior) doctor [3], with interns closely supervised by senior doctors whilst both finding their feet in the workplace and ‘sightseeing’ as they determine where they best fit in the healthcare system [4]. It is a key stage for solidifying career decisions, both of place and specialty, for many [5]. Medical graduates’ choice of internship hospital and their predominant junior doctor work location has been strongly associated with their longer-term career outcomes [6, 7]. Moreover, previous studies of rurally-based medical internships in Australia have demonstrated these to be positive and professionally satisfying experiences [8, 9]. It follows that encouraging and enabling more graduates to choose a rural internship could play a role in producing more rural doctors and partly addressing maldistribution.

Evidence supports the benefit of rural training interventions and policies for improving rural workforce supply and retention [10–12]. This evidence reinforces the positive impact of rural medical school training (the longer the better) and selecting students more likely to practice rurally because of their rural childhood origin or interest in rural work [12–17]. Rural training pathways, particularly in the early career stages, are critical interventions for producing a skilled, well-distributed and stable rural workforce. In contrast, offering financial incentives to shift the established medical workforce into relatively underserved areas have limited effect [18], and are costly [19].

Australia’s Rural Health Multidisciplinary Training program aims to improve the recruitment and retention of doctors to the rural workforce through several key initiatives, including Rural Clinical Schools (RCSs, began 2000) and Regional Training Hubs (RTHs, began late 2017) [20]. RCSs and RTHs build partnerships with medical schools, hospitals and other health services to increase clinical training and supervision capacity in rural areas, thus strengthening rural professional and social networks and career interest. Whilst RCSs have long delivered rural immersion placements for students, the newer RTHs aim to support expanded rural medical training pathways beyond medical school and starting at internship, including career guidance for rurally-interested students and junior doctors. Of note, the RCS program generally cannot support rural placements for international students within Australian medical schools whereas RTHs can support all junior doctors [21].

Outside of one Victorian study [22], there has been little published on internship preferences and acceptances, and none since the establishment of RTHs. This collaborative study focused on graduates from Queensland’s four medical schools, with James Cook University and The University of Queensland having both RCSs and RTHs, Griffith University having an RCS in partnership with Rural Medical Education Australia, and Bond University which does not have its own RCS or RTH. Queensland Health (QH) coordinates an annual internship campaign for Hospitals and Health Services (HHS) across Queensland. Similar to other States, Queensland has fewer intern positions on offer compared to the total number of eligible graduates, with an online portal used to assess applicants’ merit, compare preferences with available positions and allocate a position [23]. Eligible candidates are categorised into four groups which are in priority order from A to D, with most going through the general campaign and a small proportion through the Queensland Rural Generalist Pathway (QRGP) [24] (see Table 1). This study aims to explore preferencing and acceptance trends for rural medical internship positions in Queensland, Australia. Moreover, it focuses on internship preference and acceptance outcomes prior to and following the establishment of RTHs, and their association with key covariates such as rural training immersions offered by medical schools. A secondary aim explores factors associated with short-term rural retention in post-graduate years (PGY) 2 and 3.

**Table 1:** Applicant groups for internship allocation in Queensland, Australia

Group	Applicant eligibility	Other
A	Medical graduates who are Australian/New Zealand citizens or Australian permanent residents who have completed medical school in Queensland	Guaranteed an internship offer Can apply for internship through general pathway or Queensland Rural Generalist Pathway
B	Medical graduates who are Australian/New Zealand citizens or Australian permanent residents who have completed medical school in Australia, but not in Queensland	Not guaranteed an internship offer
C	Medical graduates of Australian universities who are NOT Australian/New Zealand citizens or Australian permanent residents	
D	Other international campuses or International medical graduates	

### Regional training hubs (RTH)

The RTH program started in late 2017, expanding to full capacity over the next 6–12 months. RTH staff are integrated within Rural Clinical Schools, focussing on supporting medical students and doctors across the medical training continuum. Each local RTH had a slightly different focus over the study period, but common activities included: (1) establishing strong relationships with all local health services, clinicians and other doctors in training including identifying (rural) career ‘champions’; (2) supporting local (rural) doctors to become clinical supervisors or improve current supervisor’s skills, including offering local skills training; (3) assisting local services in obtaining new training post accreditations; (4) supporting rurally interested students through connections to health services and clinicians, mentorship and career advice; (5) identifying and developing localised career pathway guides for relevant specialties; (6) facilitating career information sessions, intern and junior medical officer campaign webinars and sponsoring interested students to various conferences and open days. Moreover, in Queensland the two universities responsible for RTHs advocate through a joint (statewide) collaborative for regional and rural training issues at state and national levels.

## Methods

### Study design

This exploratory, descriptive study used retrospective administrative data (2014–2021 applicant rounds) of preferences and acceptances for intern positions offered by QH – the major employer of interns in Queensland, Australia. The annual intern campaign and corresponding dataset accessed for this study are managed centrally via the QH Medical Advisory & Prevocational Accreditation Unit.

Key variables accessed for this study include: Name of university; Student ID number (first linking variable, to university administrative data); Australian Health Practitioner Regulation Agency registration number (second linking variable, to publicly available practice location data); date of birth; gender; Applicant Group A-D (see Table 1); General vs QRGP candidates; location preferences (up to 20 for the general intern campaign and 14 for the QRGP); facility preference and accepted facility. Each Queensland medical school re-identified their own respective students through Student ID numbers, then matched data on rural training time, before removing identifying information and records were pooled for the analysis. Rural time was defined as four groups: 0 weeks, 1–10 weeks (those experiencing some short-term rotation/s only), 11–74 weeks (equating to 1 academic year for nearly all students) and 75 + weeks (equating to 2 or 3 academic years, where available). Across the four institutions, 6-month rotations were rarely used.

### Data entry and analysis

All data were collated in Microsoft Excel and transferred to IBM SPSS Statistics for Windows (IBM Corp, Version 22, Armonk, NY. https://www.ibm.com/analytics/spss-statistics-software) for analysis. Whilst RTHs began in late 2017, pre-RTH and post-RTH periods were defined as applicants into either 2014–18 versus 2019–2021 cohorts, with the latter corresponding with the intern allocation rounds after RTHs became operational. Group D applicants (International Medical Graduates, *n* = 60, 1%) were excluded from all analyses due to both low numbers and their negligible connection to either Queensland medical schools or RTHs.

The rurality of Queensland intern training hospitals was dichotomised, using 2019 Modified Monash Model (MM) categorisations, into metropolitan (MM-1) versus regional/rural/remote, hereafter termed ‘rural’ (MM 2–7). The respective MM classification was accessed online via ‘DoctorConnect’ (www.doctorconnect.gov.au/internet/otd/publishing.nsf/Content/locator).

**Fig. 1:**
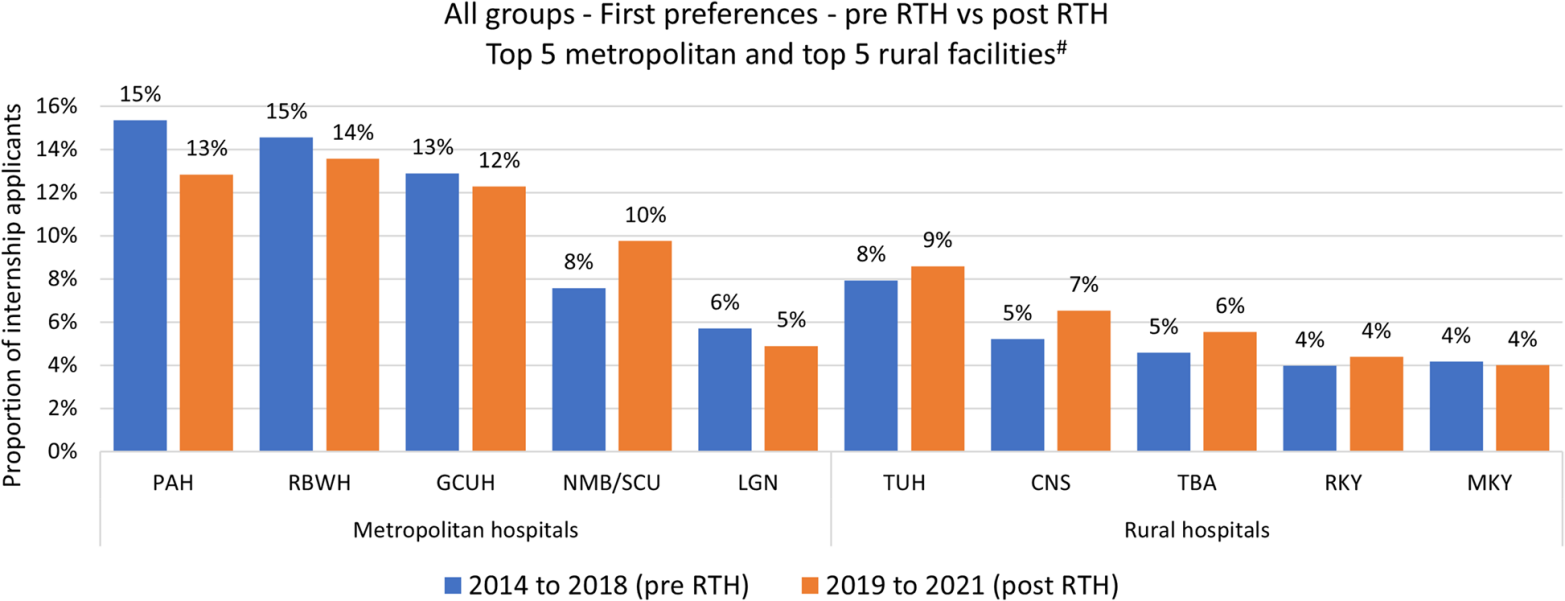
Frequency and proportion of first preferencing intern positions per hospital pre/post Regional Training Hub (RTH) program. *Facilities not shown-2014 to 2018–2019 to 2021. Other metropolitan (*n* = 7)-580 (17%)-344 (14%). Other rural (*n* = 3)-75 (3%)-66 (3%), PAH: Princess Alexandra Hospital; RBWH: Royal Brisbane and Women’s Hospital; GCUH: Gold Coast University Hospital; NMB/SCUH: Nambour Hospital & Sunshine Coast University Hospital; LGN: Logan Hospital; TUH: Townsville University Hospital; CNS: Cairns Hospital; TBA: Toowoomba Hospital; RKY: Rockhampton Hospital; MKY: Mackay Hospital

**Fig. 2:**
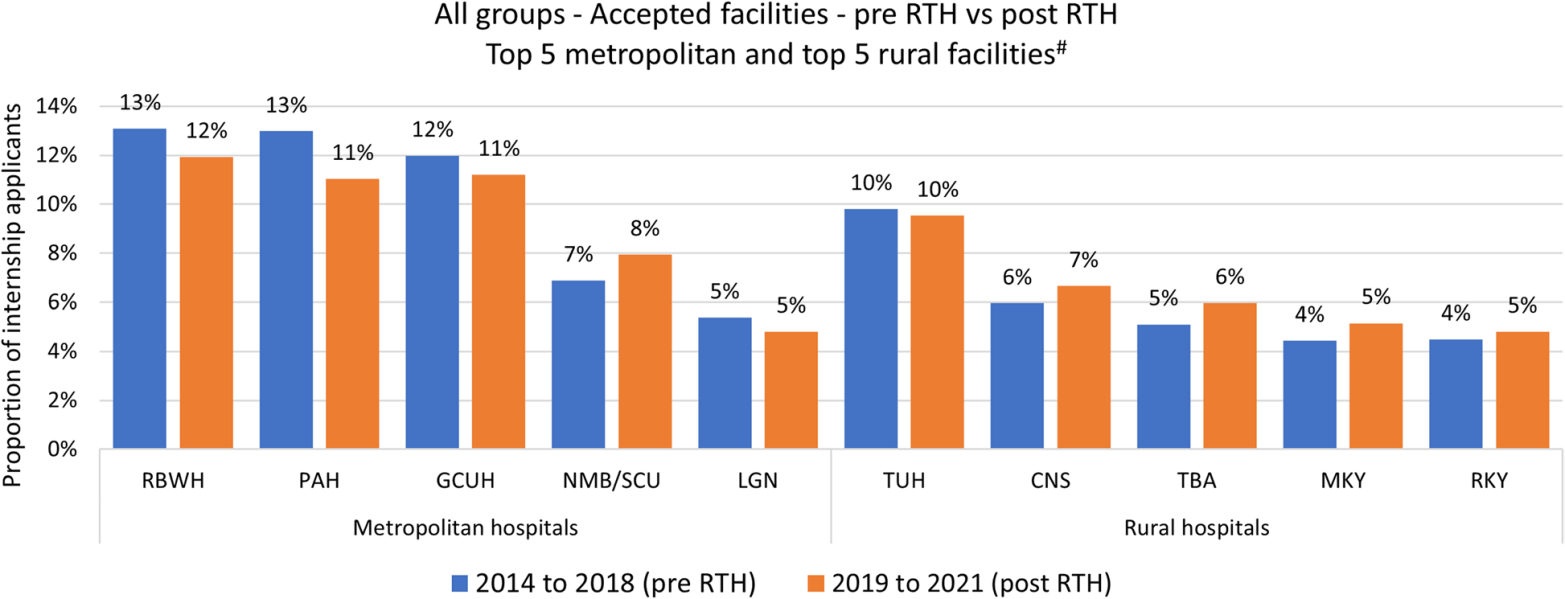
Frequency and proportion of accepted intern positions per hospital pre/post Regional Training Hub (RTH) program. *Facilities not shown, 2014 to 2018, 2019 to 2021. Other metropolitan (*n* = 7), 626 (18%), 404 (17%). Other rural (*n* = 3), 95 (3%), 83 (4%). PAH: Princess Alexandra Hospital; RBWH: Royal Brisbane and Women’s Hospital; GCUH: Gold Coast University Hospital; NMB/SCUH: Nambour Hospital & Sunshine Coast University Hospital; LGN: Logan Hospital; TUH: Townsville University Hospital; CNS: Cairns Hospital; TBA: Toowoomba Hospital; RKY: Rockhampton Hospital; MKY: Mackay Hospital

Univariate analysis determined frequency of first preferencing and accepting an intern position across all Queensland hospitals, comparing pre-RTH and post-RTH (Figs. 1 and 2). Bivariate analyses using Chi-Squared tests identified statistical associations between pre-RTH/post-RTH and key demographic, preference and acceptance variables (Table 2). Multivariable analyses (binary logistic regression) identified independent predictors for first preferencing and accepting intern positions in rural versus metropolitan hospitals (Tables 3 and 4), as well as for rural ‘retention’ (staying in MM2-7; Table 5) and ‘leaving’ metropolitan practice (moving to MM2-7; Table 6) in PGY2 and PGY3. Group B applicants (domestic graduates from a state other than Queensland) were excluded from all multivariable models as they had negligible connection to either Queensland medical schools or RTHs. The rural ‘retention’ and metropolitan ‘leaving’ analysis involved data pertaining to available years since RTHs were in place, namely 2019 and 2020 interns practising in Queensland. Level of significance was set at *p* < 0.05.

**Table 2:** Frequency and percentage of preferenced and accepted Queensland intern positions by medical graduate applicant category pre/post Regional Training Hub (RTH) program

Group A							Group B					Group C				
**Pre/ Post RTH**		**Pre-RTH** (2014-2018)		**Post-RTH** (2019-2021)		***p*-value**	**Pre-RTH** (2014-2018)		**Post-RTH** (2019-2021)		***p*-value**	**Pre-RTH** (2014-2018)		**Post-RTH** (2019-2021)		***p*-value**
**Preferenced and accepted intern positions**		**Metro**	**Non-metro**	**Metro**	**Non-metro**		**Metro**	**Non-metro**	**Metro**	**Non-metro**		**Metro**	**Non-metro**	**Metro**	**Non-metro**	
Preference 1		2373 (78.9%)	633 (21.1%)	1387 (76.0%)	439 (24.0%)	0.017	215 (69.6%)	94 (30.4%)	134 (75.3%)	44 (24.7%)	0.210	26 (9.0%)	262 (91.0%)	61 (21.1%)	228 (78.9%)	< 0.001
Preference 2		2358 (78.4%)	648 (21.6%)	1388 (76.0%)	438 (24.0%)	0.051	231 (74.8%)	78 (25.2%)	141 (79.2%)	37 (20.8%)	0.271	56 (19.4%)	232 (80.6%)	81 (28.0%)	208 (72.0%)	0.019
Preference 3		2325 (77.3%)	681 (22.7%)	1418 (77.7%)	408 (61.4%)	0.804	247 (79.9%)	62 (20.1%)	135 (75.80%)	43 (24.2%)	0.304	69 (24.0%)	219 (76.0%)	104 (36.0%)	185 (64.0%)	0.002
Preference 4		2398 (79.8%)	608 (20.2%)	1423 (77.9%)	403 (22.1%)	0.135	248 (80.3%)	61 (19.7%)	140 (78.7%)	38 (21.3%)	0.726	91 (31.6%)	197 (68.4%)	128 (44.3%)	161 (55.70)	0.002
Preference 5		2383 (79.3%)	623 (20.7%)	1454 (79.6%)	372 (20.4%)	0.769	252 (81.6%)	57 (18.4%)	139 (78.10%)	39 (21.9%)	0.408	162 (56.3%)	126 (43.8%)	150 (51.9%)	139 (48.1%)	0.316
Chose> = 2 rural facilities in the Top 5 preferences	Yes	893 (29.7%)		591 (32.4%)		0.054	94 (30.4%)		52 (29.2%)		0.837	267 (92.7%)		247 (85.5%)		0.007
	No	2113 (70.3%)		1235 (67.6%)			215 (69.6%)		126 (70.8%)			21 (7.3%)		42 (14.5%)		
Accepted intern position		2185 (72.7%)	821 (27.3%)	1283 (70.3%)	543 (29.7%)	0.070	212 (68.6%)	97 (31.4%)	134 (75.3%)	44 (24.7%)	0.121	51 (17.7%)	237 (82.3%)	76 (26.3%)	213 (73.7%)	0.016

**Table 3:** Multivariable logistic regression models for graduates from Queensland medical schools (Priority Groups A and C) choosing to first preference internship in regional or rural Queensland hospitals pre/post Regional Training Hub (RTH) program

**Predictors**	**2014 to 2018 (*n* = 2830**^b^**)**				**2019 to 2021 (*n* = 1901**^b^**)**			
	**Sample** **(*n* = 2830)**	**First preference to intern regionally (800, 28%)**	**POR** **[95%-C.I.]**^a^	***P*-value**	**Sample (*n* = 1901)**	**First preference to intern regionally (649, 34%)**	**POR** **[95%-C.I.]**^a^	***P*-** **value**
**Pathway**				< 0.001				< 0.001
General intern	2612 (92%)	646 (25%)	1		1753 (92%)	538 (31%)	1	
Rural Generalist/QRGP	218 (8%)	154 (71%)	6.7 [4.7 – 9.5]		148 (8%)	111 (75%)	6.4 [4.2 – 9.8]	
**Location in the year applying for internship**				< 0.001				< 0.001
Metropolitan (MM 1)	2094 (74%)	388 (18%)	1		1342 (71%)	327 (24%)	1	
Regional or rural (MM 2–7)	736 (26%)	412 (56%)	4.4 [3.2 – 5.9]		559 (29%)	322 (58%)	2.9 [2.1 – 4.0]	
**Time spent in undergraduate regional or rural placements for Priority Groups A and C**				< 0.001				< 0.001
Group A (0 weeks)	550 (20%)	32 (6%)	1		257 (14%)	24 (9%)	1	
Group A (1–10 weeks)	932 (33%)	58 (6%)	1.0 [0.7 – 1.6]		540 (28%)	65 (12%)	1.3 [0.8 – 2.1]	
Group A (11–74 weeks)	549 (19%)	192 (35%)	3.2 [2.1 – 5.0]		309 (16%)	83 (27%)	2.2 [1.3 – 3.6]	
Group A (75 + weeks)	520 (18%)	263 (51%)	4.4 [2.7 – 7.0]		509 (27%)	251 (49%)	3.2 [1.9 – 5.5]	
Group C	279 (10%)	255 (91%)	181.7 [104.3 – 316.7]		286 (15%)	226 (79%)	32.8 [19.6 – 54.8]	

^a^POR [95%-C.I.] = Prevalence Odds Ratio [95%-Confidence Interval]

^b^Only data of graduates with no missing values for all predictors accepted into the model were analysed (798 graduates had missing data for 1 or more predictors from 2014 to 2018, while 428 graduates had missing data for 1 or more predictors from 2019 to 2021)

**Table 4:** Multivariable logistic regression models for graduates from Queensland medical schools (Priority Groups A and C) choosing to first accept an internship in regional or rural Queensland hospitals pre/post Regional Training Hub (RTH) program

**Predictors**	**2014 to 2018 (*n* = 2830**^b^**)**				**2019 to 2021 (*n* = 1901**^b^**)**			
	**Sample** **(*n* = 2830)**	**Accept regional internship** **(939, 33%)**	**POR** **[95%-C.I.]**^**a**^	***P*-value**	**Sample (*n* = 1901)**	**Accept regional internship** **(723, 38%)**	**POR** **[95%-C.I.]**^**a**^	***P*-value**
**Pathway**				< 0.001				< 0.001
General intern	2612 (92%)	768 (29%)	1		1753 (92%)	613 (35%)	1	
Rural Generalist/QRGP	218 (8%)	171 (78%)	7.2 [5.0 – 10.4]		148 (8%)	110 (74%)	4.4 [2.9 – 6.6]	
**Location in the year applying for internship**				< 0.001				< 0.001
Metropolitan (MM 1)	2094 (74%)	488 (23%)	1		1342 (71%)	379 (28%)	1	
Regional or rural (MM 2–7)	736 (26%)	451 (61%)	3.9 [3.0 – 5.2]		559 (29%)	344 (62%)	2.9 [2.1 – 4.0]	
**Time spent in undergraduate regional or rural placements for Priority Groups A and C**				< 0.001				< 0.001
Group A (0 weeks)	550 (20%)	79 (14%)	1		257 (14%)	39 (15%)	1	
Group A (1–10 weeks)	932 (33%)	119 (13%)	0.8 [0.6 – 1.2]		540 (28%)	98 (18%)	1.2 [0.8 – 1.8]	
Group A (11–74 weeks)	549 (19%)	221 (40%)	1.6 [1.1 – 2.3]		309 (16%)	104 (34%)	1.8 [1.2 – 2.8]	
Group A (75 + weeks)	520 (18%)	291 (56%)	2.2 [1.5 – 3.2]		509 (27%)	271 (53%)	2.3 [1.4 – 3.6]	
Group C	279 (10%)	229 (82%)	27.8 [18.8 – 41.1]		286 (15%)	211 (74%)	13.7 [8.8 – 21.2]	

^a^POR [95%-C.I.] = Prevalence Odds Ratio [95%-Confidence Interval]

^b^Only data of graduates with no missing values for all predictors accepted into the model were analysed, with 798 graduates having missing data for 1 or more predictors from 2014 to 2018, and 428 graduates having missing data for 1 or more predictors from 2019 to 2021

**Table 5:** Multivariable logistic regression models for graduates from Queensland medical schools (Priority Groups A and C) remaining in regional or rural Queensland areas post-Internship

Predictor	Sample (*n* = 417)	Practising in regional or rural Queensland at PGY2 (342, 82%)	POR [95%-C.I.]^a^	*p*-value	Sample (*n* = 220)	Practising in regional or rural Queensland at PGY3 (144, 65%)	POR [95%-C.I.]^a^	*p*-value
**Priority Group**				0.038				0.002
Group C	91 (22%)	70 (77%)	1		41 (19%)	19 (46%)	19 (46%)	
Group A	326 (78%)	272 (83%)	2.0 [1.1 – 3.7]		179 (81%)	125 (70%)	125 (70%)	
**Location in the year applying for internship**				0.017				0.003
Metropolitan (MM 1)	187 (45%)	140 (75%)	1		95 (43%)	47 (50%)	1	
Regional or rural (MM 2–7)	230 (55%)	202 (88%)	1.9 [1.1 – 3.3]		125 (57%)	97 (78%)	2.6 [1.4 – 4.8]	
**First preference for internship hospital**				< 0.001				< 0.001
Metropolitan (MM 1)	76 (18%)	49 (64%)	1		47 (21%)	19 (40%)	1	
Regional or rural (MM 2–7)	341 (82%)	293 (86%)	3.7 [2.0 – 6.9]		173 (79%)	125 (72%)	4.7 [2.2 – 9.8]	

^a^POR [95%-C.I.] = Prevalence Odds Ratio [95%-Confidence Interval]

**Table 6:** Multivariable logistic regression models for graduates from Queensland medical schools (Priority Groups A and C) moving from metropolitan to regional or rural Queensland areas post-internship

Predictor	Sample (*n* = 831)	Practising in regional or rural Queensland in PGY2 (47, 6%)	POR [95%-C.I.]^a^	*p*-value	Sample (*n* = 415)	Practising in regional or rural Queensland in PGY3 (29, 7%)	POR [95%-C.I.]^a^	*p*-value
**Pathway**				0.002				0.011
General intern	804 (97%)	41 (5%)	1		402 (97%)	25 (6%)	1	
Rural Generalist/QRGP	27 (3%)	6 (22%)	6.2 [2.0 – 19.1]		13 (3%)	4 (31%)	6.3 [1.5 – 26.0]	
**First preference for internship hospital**				< 0.001				< 0.001
Metropolitan (MM 1)	784 (94%)	24 (3%)	1		392 (94%)	18 (5%)	1	
Regional or rural (MM 2–7)	47 (6%)	23 (47%)	29.9 [14.2 – 58.9]		23 (6%)	11 (48%)	18.7 [7.1 – 49.1]	

^a^POR [95%-C.I.] = Prevalence Odds Ratio [95%-Confidence Interval]

## Results

### Descriptive analysis

A total of 5956 medical graduates applied for an intern position in Queensland between 2014–2021 (61% pre-RTH and 39% post-RTH). Overall, there was a 13% (*n* = 95) increase in Queensland-based internships available per year from 2014–2021, with 63% (*n* = 60) established in rural hospitals and *n* = 35 in metropolitan hospitals (personal communication: QH). In the corresponding period, there was a 3.6% increase in total graduates from Queensland-based medical schools [25]. Queensland-trained domestic graduates (Group A) comprised the largest proportion of applicants (*n* = 4832, 81%), followed by Group C (Australian-trained international graduates, *n* = 577, 10%) and Group B applicants (interstate domestic graduates, *n* = 487, 8%).

Since establishment of the RTHs, there has been a consistent increasing trend of Australian-trained medical graduates in Queensland choosing to first preference and accept intern positions in most rural Queensland hospitals (Figs. 1 and 2). Group A applicants were significantly more likely to first preference a rural intern position post-RTH (pre 21.1% vs post 24.0%, *p* = 0.017), translating to an average additional 17 rural-based interns per year. The increase in acceptance of intern positions in rural hospitals for this group was not statistically significant (27.3% vs 29.7%, *p* = 0.07). Whilst one metropolitan facility appeared to increase its proportions (see NMB/SCUH), this corresponded with the opening of a new (and larger) hospital in 2017 in place of the previous facility. In contrast, there was a corresponding decrease in Group C applicants preferencing (91.0% vs 78.9%, *p* < 001) or accepting (82.3% vs 73.7%, *p* = 0.016) intern positions in rural hospitals between the same period. However, the actual number of Group C applicants increased over this period, and those accepting a Queensland internship increased by 66%, thus the lower acceptance rate still corresponded to a higher count (an additional 24 per year in rural hospitals).

### Multivariable analysis

Comparing 2014–18 (pre-RTH) to 2019–21 (post-RTH), the multivariable analysis showed similar predictors for both increased first preferencing and accepting intern positions in Queensland’s rural hospitals: being a Group C applicant, being a Group A applicant who spent ≥ 11 weeks training in a regional or rural town; being in the QRGP; and applying for an intern position while training in MM2-7 location (Tables 3 and 4). However, during this period, there were noticeable changes in the Prevalence Odds Ratio (POR) for graduates in both first preferencing and accepting a rural internship. Pre-RTH, students completing either 11–74 weeks or 75 + weeks placements in a MM2-7 location were more likely to accept a rural internship (POR 1.6 and 2.2, respectively) compared to those with 0 weeks rural training. Post-RTH, this likelihood increased slightly to 1.8 and 2.3, respectively. In contrast, for Group C there was a sizable decrease in likelihood of accepting a rural internship post-RTH (POR 181.7 vs 13.7), though this is counterbalanced by the sizable increase in absolute numbers (*n* = 211 over 3 years versus *n* = 229 over 5 years). It is also notable that the size of the group with 75 + rural training weeks increased by approximately 33% (from 18 to 24% of the respective cohorts) whilst maintaining a similar proportion choosing rural internships (53% versus 56%).

Table 5 identified the predictors of rural interns staying in a rural location in PGY2 (*n* = 417; 2019- 2020) and PGY3 (*n* = 220; 2019), whilst Table 6 identified predictors associated leaving metropolitan for rural practice. Between 2019 to 2021, retention of doctors in rural hospitals post-internship was 82% in PGY2 and 72% in PGY3; significant contributing factors were first preferencing a rural hospital for internship (POR = 3.7 and 4.7, respectively), being a Group A applicant (POR = 2.0 and 3.4, respectively), and training in a rural location when applying for an intern position (POR = 1.9 and 2.6, respectively). Fewer doctors moved to rural practice after a metropolitan internship; between 2019 to 2021, doctors leaving metropolitan practice for a regional hospital was 6% in PGY2 and 7% in PGY3; with ‘leaving metro’ associated with enrolment in QRGP (POR = 6.2 and 6.3, respectively) and first preferencing a regional hospital intern position. (POR = 29.9 and 18.7, respectively).

## Discussion

This study is the first to investigate possible impacts of the RTH initiative on short-term increase of the rural workforce. These results suggest a positive increase from the largest and primary target group of medical graduates (Group A, Queensland trained domestic students) choosing to both highly preference and accept an intern position in Queensland rural hospitals since RTH establishment, equating to an average 17 additions to the rural workforce each year. Findings from Group A confirm the importance of factors relating to rural connections with take-up of rural internships. Both moderate (11–74 weeks) and long-stay (75 + weeks) rural clinical training immersions were associated with preferencing rural internships, even after factoring in those who were already in a rural location during their final undergraduate year. Unsurprisingly, those entering QRGP were more likely to choose a rural intern position. These results align with previously reported Queensland medical school intention data [26, 27].

Outcomes of Group C (Australian-trained international graduates), the next largest cohort, were somewhat mixed. Proportionally, they were less likely to preference or accept a rural intern position post-RTHs; this was counterbalanced by the size of this cohort which meant the number of Group Cs working in a rural hospital also increased. Consistent with previous evidence [22], Group C applicants remained substantially more likely to both preference and accept internships in rural hospitals, which is strongly driven by the allocation process, whereby Group A and B applicant’s preferences are prioritised first. However, a common characteristic of the international students who continue working in Australia is their lack of rural connections at their time of medical school graduation, being largely excluded from government-supported rural immersions during medical school and being ineligible for the QRGP. Retention of Group C interns in rural hospitals in PGY2/3 was significantly less than Group A interns, suggesting that local integration of this group may be problematic and that the longer-term effectiveness of their current preference allocations to the rural workforce supply is weakened [21, 28]. These findings suggest longer-term rural workforce benefits from potentially expanding Australia’s Rural Health Multidisciplinary Training program to be inclusive of these students, offering QRGP beyond Group A, and preferentially selecting applicants.

Of those who completed a metropolitan internship, only a small proportion changed to a rural location within the next two years; already having a rural interest, being enrolled in the QRGP, and first preferencing a rural internship (but accepting a metropolitan internship) were the main predictors. Most notably, time spent training in a rural area and being in a rural location immediately prior to internship were not associated with returning to a rural location after their internship. This evidence confirms that the choice of internship location is a strong indication of where graduates will be in the 1–2 prevocational years that immediately follow, and thus internship is a critical point for determining workforce distribution within the early junior doctor period.

The proportion of Queensland interns experiencing extended (75 + weeks) undergraduate rural training increased greatly post-RTH and was confirmed as a significant predictor of choosing a rural internship in this study. However, the observed 33% expansion of rural placements has not led to more applicants choosing rural internships post-RTH. This suggests that even though the rural clinical training places in Queensland medical schools has significantly expanded in synergy with RTHs, more time is required to show its impact on the Queensland rural workforce.

A strength of this study was the analysis of 5956 records over 8 years representing all QH hospitals offering intern positions. However, the design of this study precludes confirming a positive impact on regional Queensland medical workforce from the establishment of RTHs, though these results are encouraging. Also, as the RTHs were operational only from late 2017 and their key activities continue to be refined, its effect may not be shown in this short study period.

The analysis did not include rural origin, a widely demonstrated factor associated with working rurally, as this measure was not wholly available for matching by all participating universities. In addition, the outbreak of the Covid-19 pandemic may have impacted relocation/immigration rates of interstate and international graduates due to travel restrictions. Finally, the quantitative nature of the study does not allow conclusions to be drawn regarding intent and drivers for accepting or staying rurally. Thus, further research using qualitative methodologies is required to investigate why medical graduates choose their internship year to be in a rural hospital and why they choose to stay or leave in PGY2 and PGY3, including hospital specific factors versus individual career interests.

## Conclusions

This study reports first evidence on the rate and trend of preferencing and uptake of rural intern positions at a whole-of-state level since establishment of RTHs. The findings show a positive association between the RTH initiative and higher preferencing of rural intern positions. However, because of the nature of the study design, it was not possible to determine if a causal relationship exists. Pleasingly, an expansion of graduates undertaking longer periods of undergraduate rural training in the same period did not diminish the proportion choosing a rural internship, suggesting there remains an appetite for more rural training opportunities. The study findings fit with existing evidence that the sustainability of rural medical workforce remains challenging, and while possibly not generalisable to other states and countries due to their context-specific features, can be used as a baseline for future research at national level to understand the impact of RTHs on addressing medical workforce distribution concerns.

## Data Availability

The datasets used and/or analysed during the current study are available from the corresponding author on reasonable request.

## Abbreviations

MM: Modified Monash (Model)
QH: Queensland Health
QRGP: Queensland Rural Generalist Pathway
PGY: Post-graduate year
POR: Prevalence Odds Ratio
RCS: Rural Clinical School
RTH: Regional Training Hub
